# Event-Based Analysis of Wildlife-Vehicle Collisions Under Temperature Extremes and Weather Variability

**DOI:** 10.3390/ani16162472

**Published:** 2026-08-08

**Authors:** Sreten Jevremović, Marko Anđelković, Aleksandra Kolarski, Filip Arnaut

**Affiliations:** 1 Institute of Physics Belgrade, National Institute of the Republic of Serbia, 11080 Belgrade, Serbia; kolarski@ipb.ac.rs (A.K.); arnaut@ipb.ac.rs (F.A.); 2 Institute for Biological Research “Siniša Stankovic”, National Institute of the Republic of Serbia, University of Belgrade, 11080 Belgrade, Serbia; marko.andjelkovic@ibiss.bg.ac.rs

**Keywords:** roadkill, temperature shocks, extreme weather conditions, Poisson regression, meteorological variability, road ecology

## Abstract

Wildlife-vehicle collisions (WVCs) represent an important ecological and road-safety problem, yet the influence of short-term meteorological variability on their occurrence remains insufficiently understood. This study investigated the associations between temperature extremes, abrupt temperature changes, and broader hydro-meteorological conditions and reported WVC frequency in Serbia over a 10-year period (2016–2025). A municipality-day dataset comprising 6281 police-reported WVCs was analyzed using an event-based methodology. Exploratory analyses were complemented by Poisson regression models to evaluate prolonged temperature episodes, abrupt temperature shocks, and broader hydro-meteorological conditions, while additional regional analyses examined the consistency of the observed associations across Serbia. The results demonstrated pronounced seasonal variation, with the highest reported WVC frequencies occurring during spring and late autumn. At the national level, prolonged cold episodes were associated with significantly lower reported WVC frequencies during their onset and middle phases, whereas heat episodes showed no significant associations. Abrupt cool-down shocks were associated with a short-term increase in reported WVC frequency on the day of the temperature decrease, while warm-up shocks showed no significant effects. Moderate and heavy precipitation, snow-day conditions, and prolonged dry spells were associated with reduced reported WVC frequency, whereas daily mean temperature and atmospheric pressure were not independently associated with reported WVC frequency. Regional analyses generally supported the national findings, although no regional associations remained statistically significant after false discovery rate correction. These findings demonstrate that short-term meteorological variability is associated with reported WVC frequency in a temporally dependent manner and highlight the importance of considering both environmental events and regional variability when investigating wildlife-vehicle collisions.

## Simple Summary

Wildlife-vehicle collisions are a growing problem because they threaten wildlife populations, endanger road users, and generate substantial economic costs. Although many studies have examined where and when these collisions occur, less is known about how short-term weather variability and extreme temperatures are associated with collision occurrence. This study analyzed police-reported wildlife-vehicle collisions across Serbia between 2016 and 2025 to investigate the effects of prolonged temperature extremes, abrupt temperature changes, precipitation, snow, and dry conditions. The results showed that reported collision frequency decreased during prolonged cold episodes, particularly during their onset and middle phases. Moderate and heavy precipitation, snow-day conditions, and prolonged dry periods were also associated with fewer reported collisions. In contrast, prolonged heat episodes showed no significant associations, while abrupt cool-down events were associated with a short-term increase in reported collision frequency on the day they occurred. Regional analyses showed that these patterns were generally consistent across Serbia, although no regional associations remained statistically significant after correction for multiple testing. These findings suggest that short-term weather variability is associated with wildlife-vehicle collisions in a temporally dependent manner and may contribute to changes in wildlife-vehicle interactions under different environmental conditions. A better understanding of these relationships can support road safety planning and wildlife conservation by identifying periods when reported wildlife-vehicle collision frequency is more likely to change.

## 1. Introduction

Wildlife-vehicle collisions (WVCs) represent one of the most widespread ecological and road-safety challenges associated with modern transportation networks [1,2]. These collisions contribute substantially to wildlife mortality, with an estimated 194 million birds and 29 million mammals killed annually on European roads alone [3]. In addition to their ecological consequences, collisions involving large mammals pose a significant risk to human safety [4,5] and generate considerable economic costs through vehicle damage, medical expenses, and traffic disruption [6,7]. As road networks continue to expand and environmental conditions become increasingly variable, understanding the factors that influence WVC occurrence has become an important objective for both transportation planning and wildlife conservation [8,9].

The occurrence of WVCs is influenced by numerous interacting factors, including wildlife abundance and behavior, traffic characteristics, road design, landscape structure, and environmental conditions [10,11]. Because these factors often operate simultaneously, understanding the contribution of individual drivers remains challenging. Spatial context, habitat characteristics, and landscape connectivity strongly influence where collisions occur, while seasonal changes in wildlife behavior, breeding, migration, and foraging determine when collision frequencies increase [12,13,14]. Furthermore, species differ considerably in their ecological requirements and behavioral responses, meaning that the influence of environmental conditions is unlikely to be uniform across wildlife communities.

Among the various factors affecting WVCs, meteorological conditions and seasonal variations have received increasing attention because they influence both wildlife activity and driving conditions [15,16,17]. Previous studies have demonstrated pronounced seasonal variation in WVC occurrence, with collision frequencies often reflecting changes in temperature, precipitation, vegetation, daylight duration, and wildlife ecology [11,18,19,20]. Long-term studies from Canada, Austria, and Brazil have shown that WVC frequencies vary substantially throughout the year, with seasonal peaks associated with increased wildlife movement and changing environmental conditions [21,22,23,24].

Weather conditions may influence WVC occurrence through several mechanisms. Temperature, precipitation, snow, and atmospheric conditions can alter wildlife movement, habitat use, visibility, road surface conditions, and driver behavior [16,17,25,26,27,28]. However, previous studies have reported inconsistent relationships. While some analyses found increased collision frequencies under warmer conditions or specific precipitation regimes [22,26], others reported reduced collision frequencies during snow, rainfall, or icy conditions, potentially reflecting changes in either wildlife activity or driver behavior [29,30]. These contrasting findings suggest that the effects of weather depend not only on the environmental conditions themselves but also on regional ecological characteristics and the temporal scale at which meteorological variability is evaluated.

Although numerous studies have examined patterns and average meteorological conditions, less attention has been devoted to discrete short-term environmental events. In particular, prolonged temperature extremes, abrupt day-to-day temperature changes, and persistent hydro-meteorological conditions such as snow or extended dry periods may trigger behavioral responses that differ from those associated with average seasonal conditions. Moreover, these different forms of meteorological variability are rarely investigated within a common analytical framework, making it difficult to compare their relative importance. Evidence from South-Eastern Europe also remains limited, despite the region’s diverse climatic conditions and growing road network.

Therefore, the aim of this paper was to investigate the influence of short-term meteorological variability on recorded WVCs in Serbia during the period 2016–2025. Specifically, the study examined the effects of prolonged heat and cold episodes, the effects of abrupt day-to-day temperature changes, and the effects of broader hydro-meteorological conditions, including precipitation, snow-day conditions, and dry spells. In addition, regional analyses were performed to evaluate whether these relationships differed among the major geographic regions of Serbia. By integrating these analyses within a unified statistical framework, this study provides an assessment of how different forms of short-term weather variability are associated with recorded WVC occurrence.

## 2. Materials and Methods

### 2.1. Study Area and Data Sources

The research was conducted in the Republic of Serbia, a country located in the central Balkan Peninsula characterized by considerable climatic and topographic heterogeneity. Northern Serbia (Vojvodina) is predominantly lowland with a temperate continental climate, whereas central and southern parts of the country are hilly and mountainous with greater climatic variability. The climate is characterized by warm summers and cold winters, with mean annual air temperatures ranging from approximately 10 to 13 °C depending on elevation and region. July and August are generally the warmest months, while January is the coldest. Mean annual precipitation varies from approximately 550 mm in northern areas to more than 1000 mm in mountainous regions [31].

The analysis covered the period from 1 January 2016 to 31 December 2025. Wildlife-vehicle collision records were obtained from the Ministry of Interior of the Republic of Serbia [32] and included only police-reported traffic accidents. Following spatial processing in a Geographic Information System (GIS), only collisions occurring on rural roads were retained, while urban-road collisions were excluded from further analyses. Because collisions involving small animals frequently remain unreported, the database primarily represents accidents involving medium-sized and large wildlife that resulted in official police records. The most common medium- and large-sized mammal species occurring in Serbia include roe deer, wild boar, red fox, jackal, European hare, and European badger, although species-level information was not available in the collision database [33].

Daily meteorological data were obtained from the NASA POWER database v2.6.13 [34] for each municipality. The initial meteorological dataset included daily mean air temperature (°C), total precipitation (mm), daily mean atmospheric pressure (kPa), and daily mean relative humidity (%). These variables were selected because they represent the principal thermal and hydro-meteorological conditions examined in this study.

For the regional analyses, Serbia was divided according to the NUTS-2 classification into four regions: Vojvodina, Belgrade, Central and Western Serbia, and Southern and Eastern Serbia [35].

### 2.2. Data Preprocessing

#### 2.2.1. Preprocessing

Before the statistical analyses, the WVC and meteorological datasets were prepared and merged into a single database. Municipality names and temporal variables were standardized, duplicate records were checked, and the consistency of municipality-date combinations was verified. Daily meteorological observations were assigned to municipalities and merged with the collision records to create a municipality-day dataset, in which each record represented one municipality on one calendar day. Because regression analyses were performed at the municipality-day level, days without reported WVCs were retained in the dataset and coded as zero. These observations represent the absence of a reported WVC rather than missing data and were required to estimate daily associations between meteorological conditions and reported WVC frequency across all municipalities and throughout the entire study period. The final national dataset comprised 91,325 municipality-day observations from 25 administrative areas over the study period, including 6281 reported WVCs. Most municipality-days (93.7%) contained no reported collisions, whereas 6.3% contained one or more recorded WVCs.

For the regional analyses, municipalities were grouped into four broader geographic regions of Serbia based on the NUTS-2 classification [35]: Vojvodina, Belgrade, Central and Western Serbia, and Southern and Eastern Serbia. Separate municipality-day datasets were then generated for each region while preserving the same data structure and preprocessing procedure used for the national analyses.

Descriptive statistics were calculated to summarize the characteristics of the whole dataset and the distributions of both WVCs and meteorological variables. Pearson correlation coefficients were subsequently calculated among the continuous meteorological variables to evaluate potential multicollinearity before regression modeling. Because air temperature and relative humidity exhibited a strong correlation, relative humidity was excluded from subsequent regression analyses, while air temperature was retained as the indicator of thermal conditions.

#### 2.2.2. Temperature Episodes

Temperature episodes were defined to identify sustained periods of unusually high or low air temperatures. To account for regional climatic differences, all thresholds were calculated separately for each municipality using municipality temperature distributions.

Heat episodes were identified during the summer period (June–August) as periods of at least two consecutive days with mean daily air temperatures exceeding the 95th percentile of the corresponding municipality. Cold episodes were identified during the winter period (December–February) as periods of at least two consecutive days with mean daily air temperatures below the 5th percentile.

Each temperature episode was divided into five mutually exclusive temporal phases describing its progression: onset, middle, end, first day after, and second day after the episode. The onset and end phases each consisted of a single day, corresponding to the first and last day of the episode, respectively. For episodes lasting more than two days, all intermediate days were classified as the middle phase. The first and second day after the episode represented the first and second day immediately following the end of each episode, respectively.

Separate binary indicator variables were created for each episode phase, with all municipality-days not belonging to the corresponding phase serving as the reference category. These indicators were subsequently used to evaluate whether reported WVC frequency differed during specific stages of prolonged heat and cold episodes.

#### 2.2.3. Temperature Shocks

Temperature shocks were defined to identify abrupt day-to-day changes in mean daily air temperature. Similar to the temperature episodes, all thresholds were calculated separately for each municipality to account for local climatic conditions.

Warm-up shocks were defined as days when the increase in mean daily air temperature compared with the previous day exceeded the 95th percentile of all positive day-to-day temperature changes for the corresponding municipality. Cool-down shocks were defined as days when the decrease in mean daily air temperature exceeded the 95th percentile of all negative day-to-day temperature changes within the same municipality.

For each shock type, three mutually exclusive temporal phases were defined: same day, first day after, and second day after the shock event. The same-day phase represented the day on which the abrupt temperature change occurred, whereas the first- and second-day phases represented the first and second day following the event, respectively.

Separate binary indicator variables were created for each shock phase, with all municipality-days not belonging to the corresponding phase serving as the reference category. These indicators were subsequently used to evaluate whether reported WVC frequency differed immediately following abrupt increases or decreases in daily air temperature.

#### 2.2.4. Broader Hydro-Meteorological Variables

The hydro-meteorological analyses included both continuous and categorical environmental variables derived from the daily meteorological data. The continuous variables comprised mean daily air temperature, total daily precipitation, mean daily atmospheric pressure, and mean daily relative humidity.

Daily precipitation was additionally classified into four categories: no precipitation, light (0–5 mm), moderate (5–20 mm), and heavy (>20 mm). Snow-day conditions were represented using a proxy defined as days with precipitation and a mean daily air temperature ≤ 0 °C. Consecutive days without precipitation were used to define dry-spell duration, which was categorized as short (0–2 consecutive dry days), moderate (3–6 consecutive dry days), and prolonged (≥7 consecutive dry days). Prior to the statistical analyses, Pearson correlation coefficients were calculated among the continuous meteorological variables to evaluate potential multicollinearity.

### 2.3. Statistical Analysis

#### 2.3.1. National and Regional Analysis

The outcome variable consisted of the daily number of reported WVCs recorded within each municipality. Because the response variable represented count data, Poisson and Negative Binomial regression models were initially evaluated. All regression models included municipality, month, day of the week, and year as fixed effects to account for spatial heterogeneity, seasonal variation, weekly reporting patterns, and long-term temporal trends. Model adequacy was assessed using goodness-of-fit statistics, dispersion diagnostics, residual analyses, zero-frequency assessment, Ljung–Box tests of temporal autocorrelation in deviance, Pearson, and randomized quantile residuals, and sensitivity analyses involving alternative count-model specifications. The results of the model-selection procedure, including comparisons between Poisson and Negative Binomial regression models, diagnostic and other statistics, are presented in the Supplementary Materials (Tables S12–S14). Based on these evaluations, Poisson regression provided the most appropriate balance between model fit, model assumptions, and interpretability and was therefore adopted for all subsequent analyses.

Municipality fixed effects were implemented as indicator variables, while month, day of the week, and year were included as categorical predictors. The same regression framework was applied throughout the study. However, the set of explanatory variables differed according to the specific research question. Temperature episode and temperature shock models included precipitation and atmospheric pressure as adjustment covariates to reduce potential confounding, whereas hydro-meteorological models evaluated these variables as predictors of interest. The specific model specifications for each analysis are described in the following subsections. Model results are presented as incidence rate ratios (IRRs) with corresponding 95% confidence intervals.

The primary analyses were performed using the national municipality-day dataset. To evaluate whether the observed national associations were consistent across Serbia, additional analyses were performed separately for four geographic regions derived from the NUTS-2. The same modeling framework and adjustment variables were retained for both the national and regional analyses to ensure direct comparability of the estimated associations. Because multiple analyses were performed across the regional analyses, *p*-values were additionally adjusted using the Benjamini–Hochberg false discovery rate (FDR) procedure.

Exploratory descriptive analyses were also performed to characterize seasonal and annual patterns of reported WVCs, as well as event temporal trends. All statistical analyses were performed in Python 3.10.

#### 2.3.2. Temperature Episodes

The associations between prolonged temperature episodes and reported WVC frequency were evaluated using the general Poisson regression framework described in the previous section. Separate regression models were fitted for heat and cold episodes, with the corresponding episode-phase indicator variables included as the explanatory variables of interest.

For each model, municipality, month, day of the week, and year were included as fixed effects, while daily precipitation and atmospheric pressure were incorporated as adjustment covariates to reduce potential confounding. Each episode phase (onset, middle, end, first day after, and second day after) was evaluated separately by comparing municipality-days belonging to the corresponding phase with all remaining municipality-days.

Model estimates are presented as IRRs with corresponding 95% confidence intervals. Statistical significance was evaluated at the 0.05 level.

#### 2.3.3. Temperature Shocks

The associations between abrupt temperature shocks and reported WVC frequency were also evaluated using the general Poisson regression framework. Separate regression models were fitted for warm-up and cool-down shocks, with the corresponding shock-phase indicator variables included as the explanatory variables of interest.

For each model, municipality, month, day of the week, and year were included as fixed effects, while daily precipitation and atmospheric pressure were incorporated as adjustment covariates to reduce potential confounding. Each shock phase (same day, first day after, and second day after) was evaluated separately by comparing municipality-days belonging to the corresponding phase with all remaining municipality-days.

Model estimates are presented as IRRs with corresponding 95% confidence intervals. Statistical significance was evaluated at the 0.05 level.

#### 2.3.4. Broader Hydro-Meteorological Analysis

As in the previous subchapters, the associations between hydro-meteorological conditions and reported WVC frequency were evaluated using the general Poisson regression framework. The explanatory variables included precipitation category, snow-day conditions, dry-spell duration, mean daily air temperature, and atmospheric pressure.

Municipality, month, day of the week, and year were included as fixed effects in all models to account for spatial heterogeneity, seasonal variation, weekly reporting patterns, and long-term temporal trends. Unlike the temperature episode and temperature shock analyses, precipitation and atmospheric pressure were evaluated as predictors of interest rather than adjustment covariates. Relative humidity was not included in the regression models because it exhibited strong collinearity with mean daily air temperature.

Model estimates are presented as IRRs with corresponding 95% confidence intervals. Statistical significance was evaluated at the 0.05 level.

## 3. Results

### 3.1. Descriptive Analyses

The final sample of 6281 police-reported wildlife-vehicle collisions was used in this paper. Most municipality-days (93.7%) contained no reported collisions, resulting in a sparse count distribution with a median daily WVC count of zero. Mean daily air temperature during the study period was 12.22 ± 9.33 °C, while precipitation exhibited a highly skewed distribution characterized by many dry days. Atmospheric pressure showed relatively low day-to-day variability throughout the study period. A summary of the descriptive statistics for all variables included in the regression analyses is presented in Table 1.

#### 3.1.1. National Seasonal Patterns

**Figure 1:**
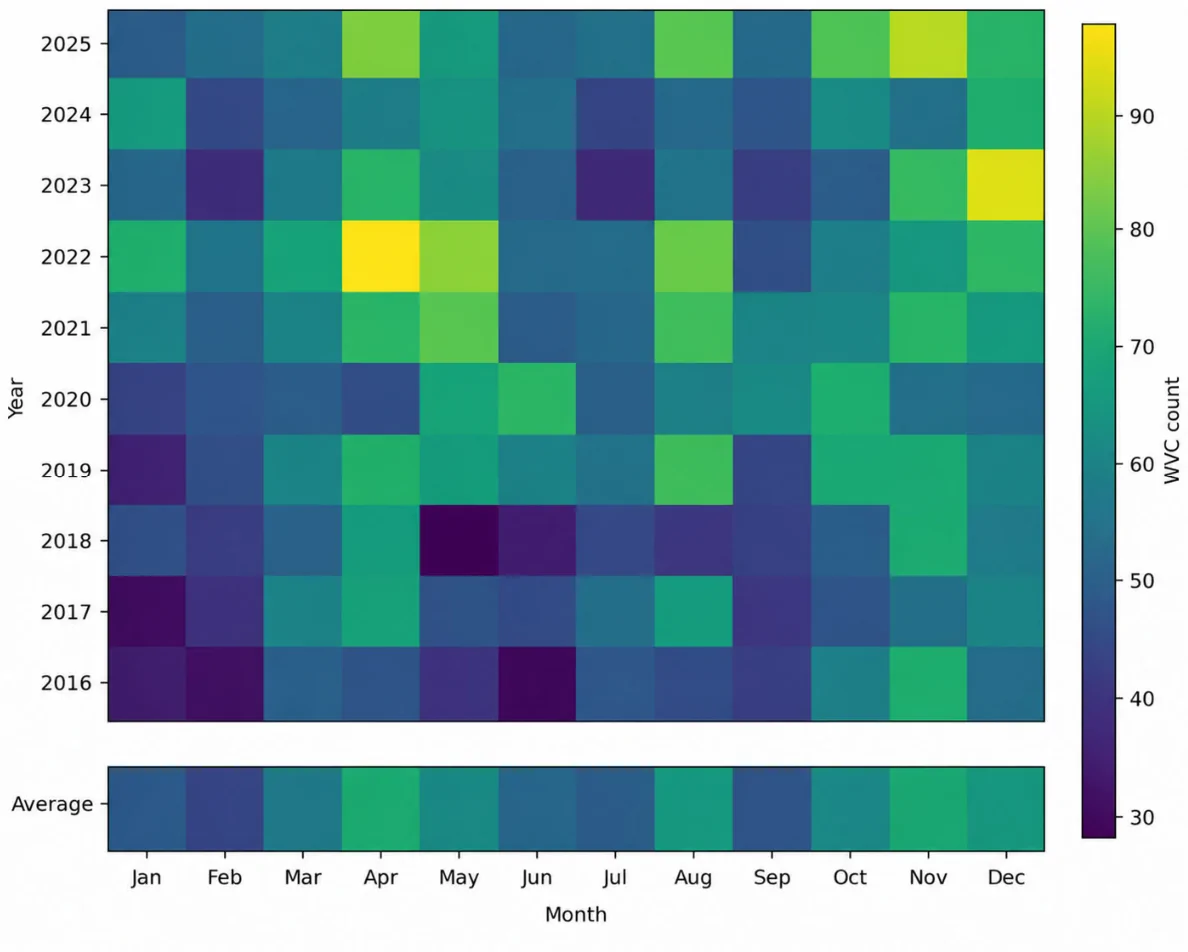
Monthly distribution of WVCs in Serbia during 2016–2025.

Monthly variation in reported WVCs was initially examined to characterize the seasonal distribution of WVC occurrence throughout the study period. As shown in Figure 1, reported WVC frequencies showed a clear and consistent seasonal pattern across the 2016–2025 period. Collision frequencies were generally highest during spring and late autumn, with pronounced peaks in April, whereas the lowest numbers of reported collisions were observed during the winter months, particularly January and February. Although the magnitude of monthly counts varied among years, the overall seasonal pattern remained relatively stable.

To evaluate these temporal differences, month was included as a categorical predictor in the final Poisson regression model while controlling for municipality, year, and day-of-week effects. The results confirmed significant seasonal variation in reported WVC frequency (Table 2, Supplementary Figure S1). Compared with January, significantly higher collision frequencies were observed in March, April, May, August, October, November, and December, whereas February, June, July, and September did not differ significantly from the reference month. The largest increases in reported WVC frequency were estimated for April and November, confirming these months as the primary seasonal peaks in WVC occurrence across Serbia.

#### 3.1.2. Regional Seasonal Patterns

**Figure 2:**
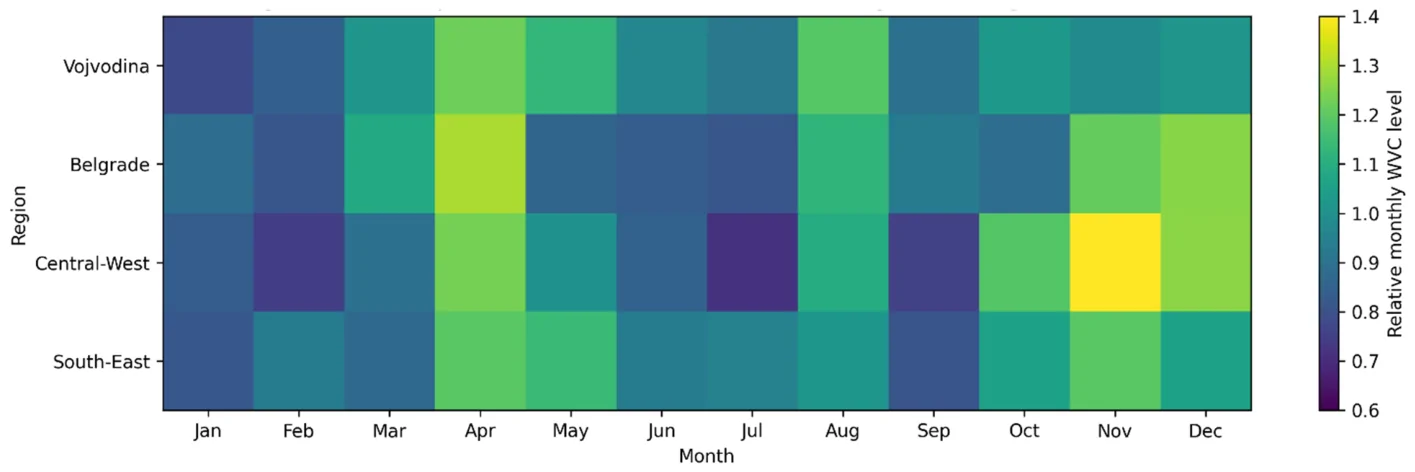
Regional seasonal patterns of WVCs in Serbia during 2016–2025 (values above 1 indicate above-average collision frequencies and values below 1 indicate below-average frequencies).

In order to determine whether the seasonal pattern observed at the national level was consistent across Serbia, monthly WVC frequencies were examined separately for each of the four study regions. Monthly collision frequencies were standardized relative to the regional monthly mean to facilitate comparison among regions with different overall collision levels (Figure 2).

All four regions exhibited a broadly similar seasonal structure, characterized by increased reported WVC frequencies during spring and late autumn. April consistently represented one of the months with the highest collision frequencies across all regions, while elevated WVC occurrence was also observed during November in most regions. In contrast, the lowest frequencies were generally recorded during January and February. Although the timing of the principal seasonal peaks remained largely consistent, their magnitude varied among regions, indicating that local environmental and ecological conditions may influence the strength of seasonal variation.

These observations were confirmed by the regional Poisson regression analyses (Table 3), which demonstrated that the seasons associated with significantly elevated reported WVC frequencies differed somewhat among regions, although the general seasonal pattern remained comparable throughout Serbia. The Belgrade region exhibited substantially narrower confidence intervals than the other regions (Belgrade was analyzed as a single administrative unit, unlike the other regions that comprised multiple administrative areas), reflecting the higher precision of the regional estimates rather than stronger seasonal effects. Overall, the results indicate that the national seasonal pattern is representative of all four study regions, with regional differences primarily reflecting variation in the magnitude rather than the timing of seasonal changes.

### 3.2. Temperature Episodes

#### 3.2.1. National Analysis

**Figure 3:**
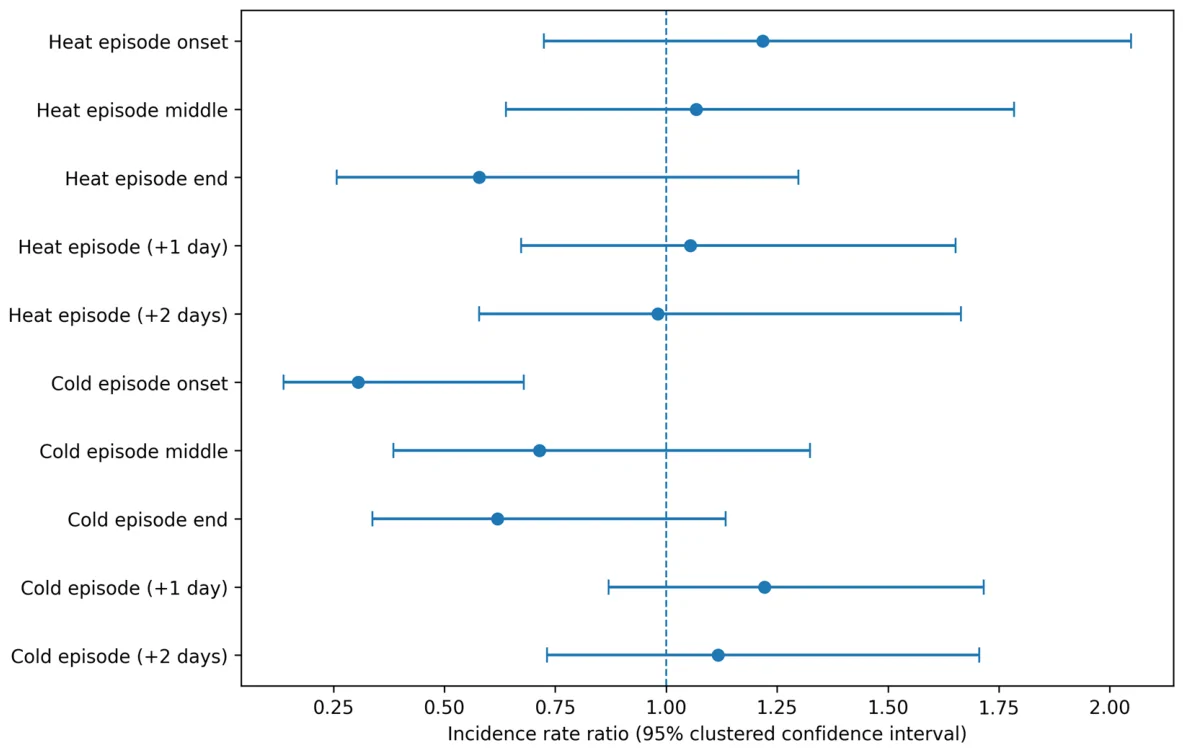
Associations between temperature-episode phases and reported WVC frequency estimated using the Poisson regression model.

The estimated IRRs and corresponding 95% confidence intervals for each episode phase are presented in Figure 3, while the results of the analysis in more detail are presented in the Supplementary Materials (Tables S1 and S2).

Heat episodes showed no statistically significant association with reported WVC frequency during any of the analyzed phases, indicating that prolonged periods of unusually high temperatures did not substantially influence reported WVC occurrence.

In contrast, cold episodes exhibited a clear temporal pattern. The onset of cold episodes was associated with a significant reduction in reported WVC frequency, and this decrease persisted during the middle phase of the episode. However, no significant effects were observed during the end of cold episodes or during the first and second post-episode days, indicating that the reduction in reported WVC frequency was confined to the early stages of prolonged cold conditions.

#### 3.2.2. Regional Analysis

**Figure 4:**
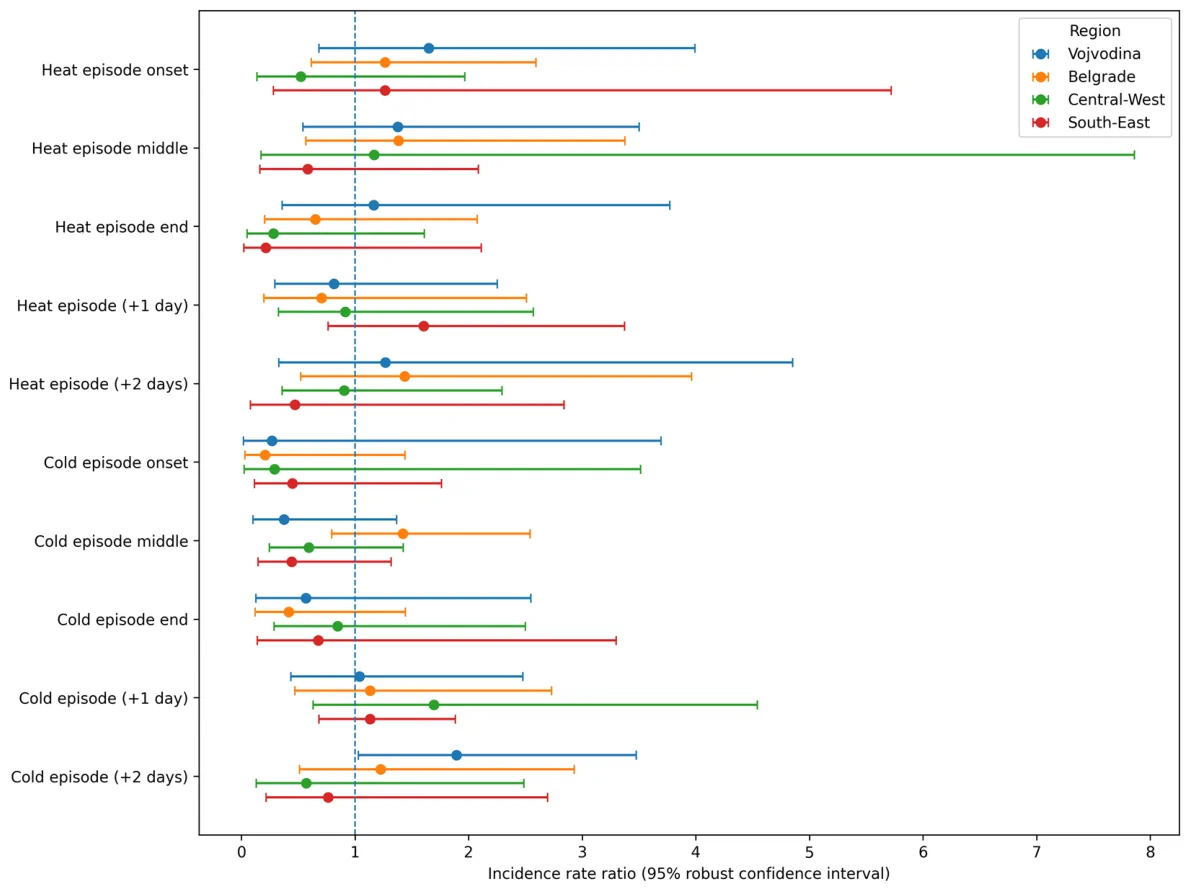
Regional associations between temperature-episode phases and reported WVC frequency estimated using Poisson regression models.

The estimated IRRs and corresponding 95% confidence intervals are presented in Figure 4, while the complete regional regression results, including false discovery rate *p*-values, are provided in the Supplementary Materials (Table S9).

The regional analyses generally showed effect estimates that were consistent with the national findings, although the magnitude of the associations varied among regions. Heat episodes did not exhibit consistent regional effects, with all estimated associations remaining statistically non-significant. For cold episodes, several regions showed trends in the same direction as the national analysis, particularly during the onset and middle phases, although these associations did not reach statistical significance. The only nominally significant regional association was observed for the second day following cold episodes in Vojvodina, where reported WVC frequency was higher than during non-episode days. However, this association was no longer statistically significant after applying the FDR correction for multiple comparisons (Table S9).

This regional analysis did not identify statistically robust regional differences in the effects of temperature episodes after accounting for multiple testing. Nevertheless, the similarity in the direction of several regional effect estimates suggests that the national patterns were broadly consistent across Serbia, although the strength of these associations varied among regions.

### 3.3. Temperature Shocks

#### 3.3.1. National Analysis

**Figure 5:**
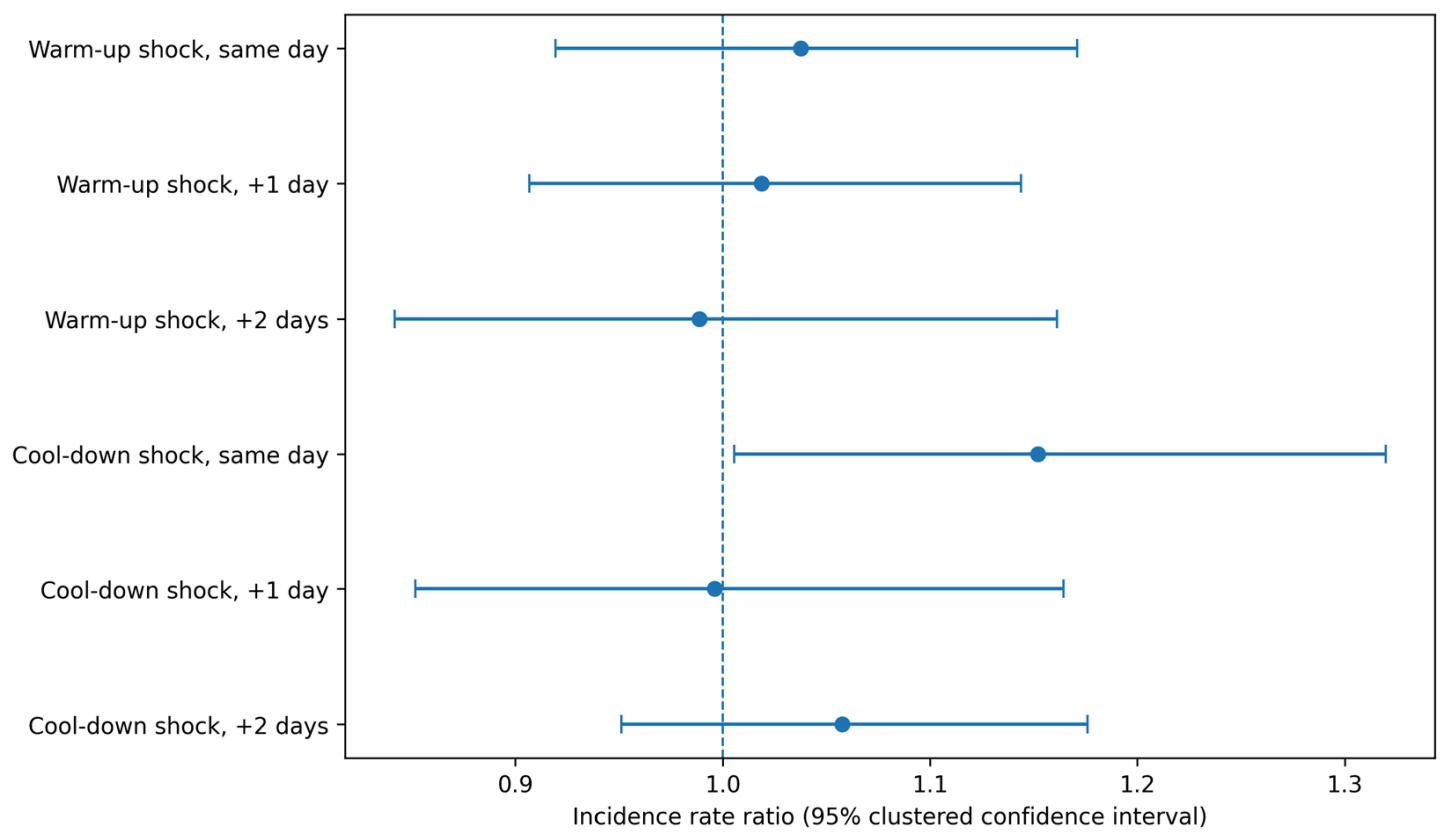
Associations between temperature-shock phases and reported WVC frequency estimated using the Poisson regression model.

The estimated IRRs and corresponding 95% confidence intervals for each temperature-shock phase are presented in Figure 5, while the more detailed results are provided in the Supplementary Materials Tables S3 and S4.

Warm-up shocks were not associated with significant changes in reported WVC frequency. In contrast, cool-down shocks were associated with a significant increase in reported WVC frequency on the day of the temperature decrease (IRR = 1.15, 95% CI: 1.01–1.32), whereas no significant effects were observed one or two days after the shock.

#### 3.3.2. Regional Analysis

**Figure 6:**
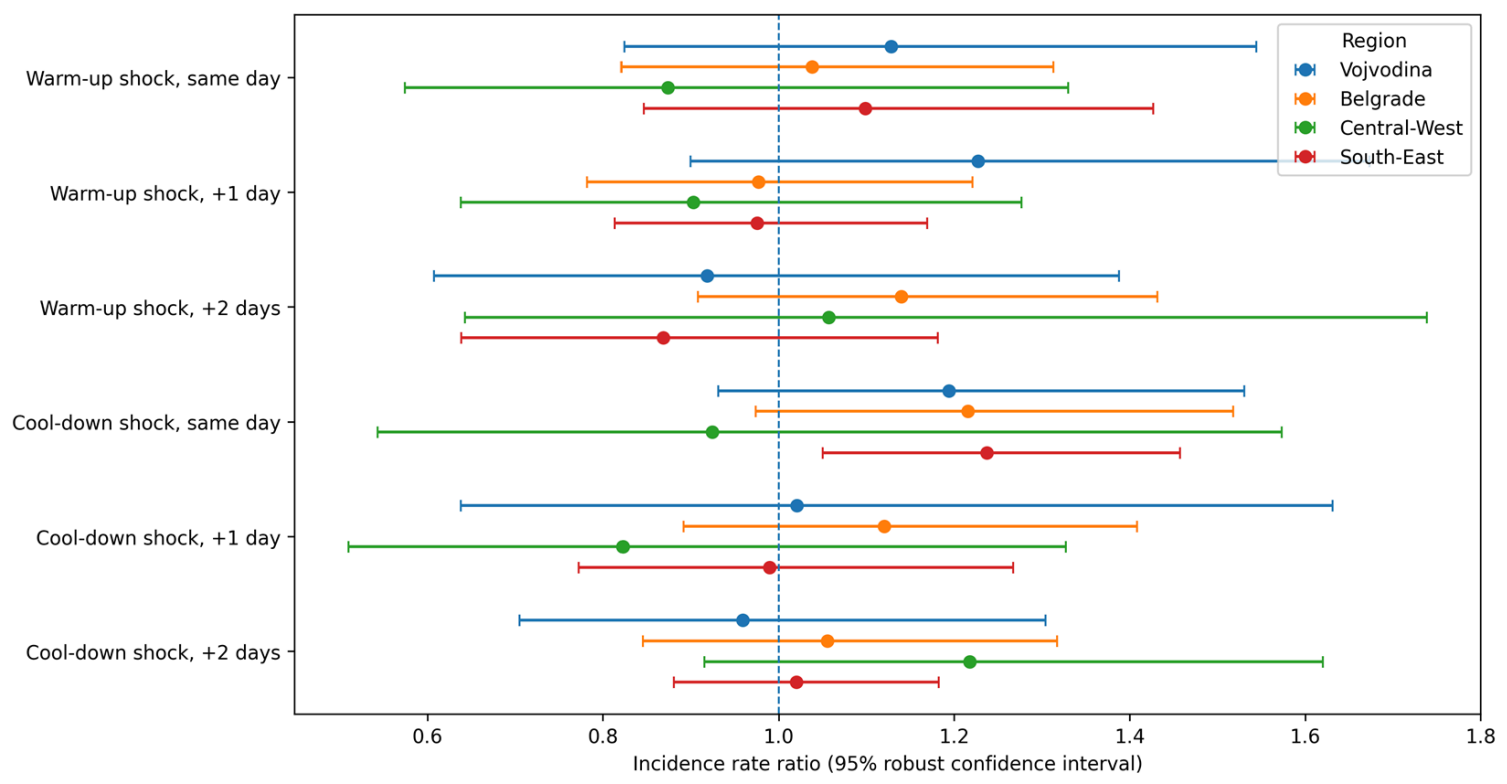
Regional associations between temperature-shock phases and reported WVC frequency estimated using Poisson regression models.

The temperature-shock analyses were repeated separately for each of the four study regions. The estimated IRRs and corresponding 95% robust confidence intervals are presented in Figure 6, while the complete regional regression results, including FDR *p*-values, are provided in Supplementary Materials (Table S10).

The regional analyses showed considerable uncertainty in the estimated effects of both warm-up and cool-down shocks, as reflected by the relatively wide confidence intervals. This was expected because the regional models were based on substantially fewer observations and event days than the national analysis, resulting in lower statistical precision. Overall, no consistent regional associations between temperature shocks and reported WVC frequency were identified. The only nominally significant association was observed for same-day cool-down shocks in the Southeast region, where reported WVC frequency was higher than during non-shock days. However, this association was no longer statistically significant after applying the FDR correction for multiple testing (Table S10).

The regional analyses did not provide evidence for statistically robust regional differences in the effects of temperature shocks after accounting for multiple comparisons. Although several regional effect estimates differed in magnitude, the broad overlap of the confidence intervals indicates that the observed variation most likely reflects the limited precision of the regional estimates rather than consistent differences among regions.

### 3.4. Broader Hydro-Meteorological Analysis

#### 3.4.1. National Analysis

**Figure 7:**
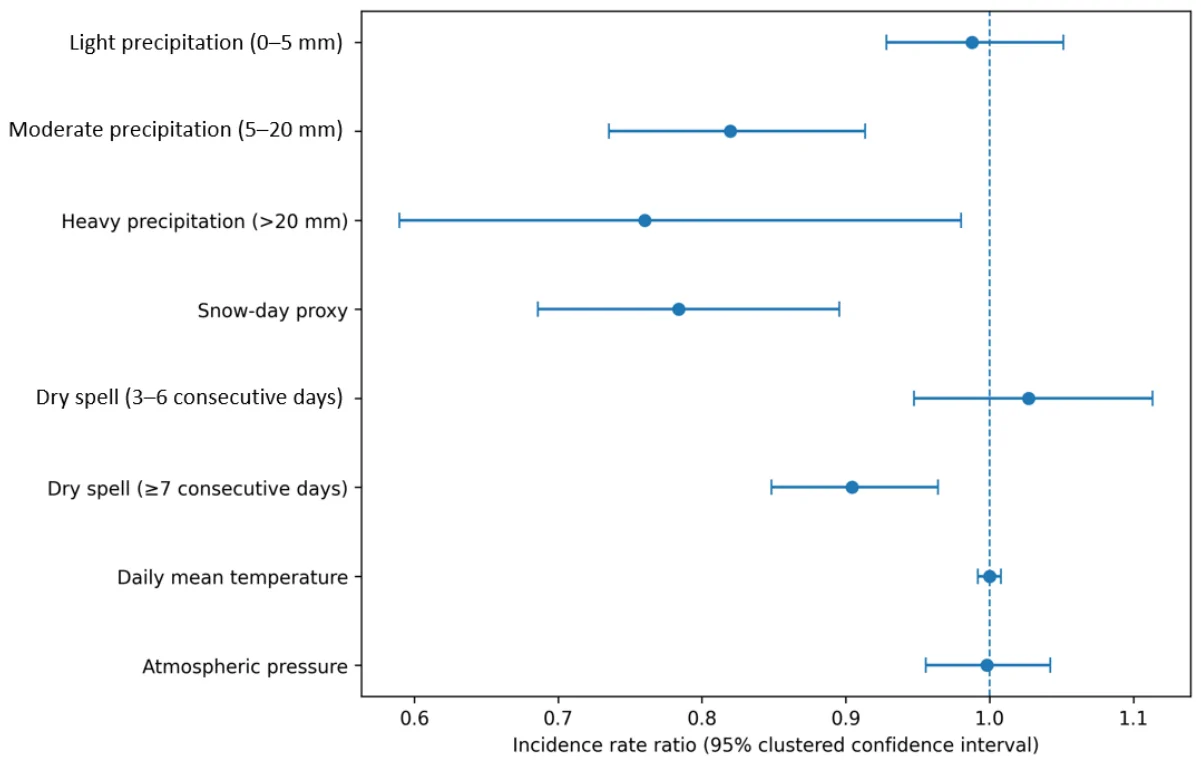
Associations between hydro-meteorological conditions and reported WVC frequency estimated using the Poisson regression model.

Estimated IRRs and their corresponding 95% confidence intervals are presented in Figure 7, while the complete regression results are provided in Supplementary Materials (Tables S5 and S6).

Moderate precipitation was associated with an approximately 18% reduction in reported WVC frequency (IRR = 0.82, 95% CI: 0.74–0.91), while heavy precipitation was associated with an approximately 24% reduction (IRR = 0.76, 95% CI: 0.59–0.98). The snow-day proxy also showed a significant negative association, corresponding to an approximately 22% lower reported WVC frequency compared with non-snow days (IRR = 0.78, 95% CI: 0.69–0.90).

Prolonged dry spells lasting at least seven consecutive days were associated with an approximately 10% reduction in reported WVC frequency (IRR = 0.90, 95% CI: 0.85–0.96), whereas dry spells lasting three to six days were not significant. Light precipitation, daily mean temperature, and atmospheric pressure were also not significantly associated with reported WVC frequency.

#### 3.4.2. Regional Analysis

**Figure 8:**
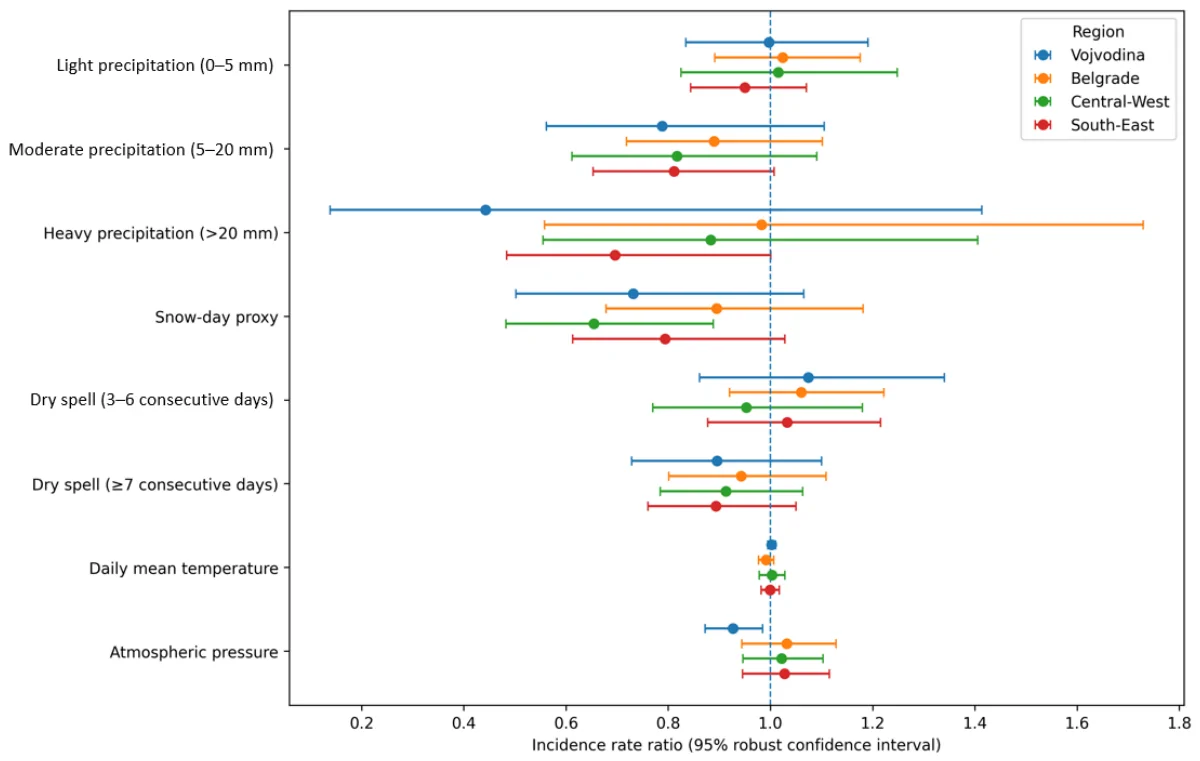
Regional associations between hydro-meteorological conditions and reported WVC frequency estimated using Poisson regression models.

The estimated IRRs and corresponding 95% confidence intervals are presented in Figure 8, while the complete regional regression results, including FDR *p*-values, are provided in Supplementary Materials (Table S11).

The regional analyses generally showed effect estimates that were consistent with the national analysis, with moderate and heavy precipitation, snow-day conditions, and prolonged dry spells tending to be associated with lower reported WVC frequencies. However, the magnitude of these associations varied among regions and was accompanied by relatively wide confidence intervals, reflecting the smaller number of observations and event days available for the regional models compared with the national analysis.

Two associations reached nominal statistical significance before correction for multiple testing: a negative association between snow-day conditions and reported WVC frequency in the Central West region and a negative association between atmospheric pressure and reported WVC frequency in Vojvodina. However, neither association remained statistically significant after applying the FDR correction (Tables S7, S8 and S11).

The regional analyses did not provide evidence for statistically robust regional differences in the effects of hydro-meteorological conditions after accounting for multiple comparisons. Nevertheless, the broadly similar direction of the regional effect estimates supports the conclusion that the national associations are generally representative across Serbia, while regional differences primarily reflect the lower precision of the regional estimates rather than systematic geographic variation.

## 4. Discussion

This paper examined the associations between temperature extremes, abrupt temperature changes, and broader hydro-meteorological conditions and reported WVC frequency across Serbia. The results show that meteorological conditions were associated with the reported WVC frequency in a complex and temporally dependent manner. The strongest and most consistent associations were observed for prolonged cold episodes and selected hydro-meteorological conditions, whereas heat episodes and abrupt temperature changes showed comparatively limited effects. In addition, the analyses revealed pronounced seasonal variation, with elevated reported WVC frequencies during spring and late autumn, indicating that short-term meteorological influences occur within broader seasonal patterns. These findings support previous research suggesting that weather conditions are associated with WVCs through multiple interacting ecological and behavioral pathways rather than through simple direct effects of individual meteorological variables [36]. Although the magnitude of the estimated associations varied among regions, the regional analyses generally showed patterns consistent with the national analyses, suggesting that the observed relationships were relatively robust across different parts of Serbia while also highlighting the importance of considering regional heterogeneity when interpreting local patterns.

The pronounced seasonal pattern observed in this study is consistent with previous research demonstrating that WVC occurrence is closely linked to annual cycles of wildlife activity [11,22,24]. Reported WVC frequencies were highest during spring and late autumn, with the strongest increases occurring in April and November, while similar temporal patterns were observed across all four study regions. The consistency of these seasonal patterns suggests that short-term meteorological influences should be interpreted within a broader seasonal context, in which weather conditions may modify the timing or intensity of wildlife movements rather than determine their overall occurrence. Previous studies have attributed seasonal peaks in WVC frequency to periods of increased wildlife movement associated with reproduction, foraging, and seasonal habitat shifts [11,22,24,37,38,39]. However, because species-level information was not available in the present study, these mechanisms should be considered as possible explanations rather than direct drivers of the observed seasonal patterns. The broadly similar seasonal trends identified across all analyzed regions further suggest that these temporal patterns are relatively consistent throughout Serbia, although the magnitude of weather-related associations may vary among regions.

The present study further showed that the duration and persistence of temperature extremes are important when evaluating their associations with reported WVC frequency. In contrast to heat episodes, which exhibited weak and inconsistent associations, prolonged cold episodes were associated with lower reported WVC frequencies, particularly during their onset and middle phases. This finding is consistent with previous studies suggesting that extended periods of low temperatures may reduce animal activity, limit movement, and consequently decrease the likelihood of wildlife encountering roads [19,24]. However, these mechanisms should be regarded as plausible explanations rather than direct evidence, since neither wildlife movement nor traffic volume was measured in the present study. Reduced reported WVC frequency during prolonged cold periods may also reflect changes in driver behavior, lower traffic volumes during adverse weather, or a combination of these factors. In contrast, prolonged heat episodes were not associated with consistent changes in reported WVC frequency, suggesting that elevated temperatures alone may have a more limited influence on reported collisions under the environmental conditions represented in this study. Although the underlying reasons remain uncertain, this difference may indicate that wildlife responses to prolonged cold and heat conditions are not equivalent.

The results identified contrasting associations between prolonged cold episodes and abrupt cool-down shocks. Whereas prolonged cold episodes were associated with lower reported WVC frequency, same-day cool-down shocks were associated with a temporary increase in reported WVC frequency. Although these findings may initially appear contradictory, they likely reflect different patterns of short-term temperature variability. Cold episodes represent several consecutive days of persistently low temperatures, whereas cool-down shocks represent abrupt day-to-day decreases in temperature. One possible explanation is that sudden temperature declines temporarily alter wildlife movement or driver behavior before animals and road users adjust to the new environmental conditions. In contrast, once cold conditions persist over several days, wildlife activity, traffic volume, or both may decrease, resulting in fewer reported WVCs. The findings suggest that the duration and rate of temperature change may be as important as the absolute weather conditions when evaluating associations between meteorological conditions and reported WVC frequency. This distinction has received relatively little attention in previous WVC research and suggests that future studies should consider both persistent weather episodes and abrupt temperature changes rather than relying solely on daily meteorological variables.

The study also demonstrated that several broader hydro-meteorological conditions were associated with reported WVC frequency. Moderate and heavy precipitation, snow-day conditions, and prolonged dry spells were associated with lower reported WVC frequencies, whereas light precipitation, daily mean temperature, and atmospheric pressure showed no significant independent associations. These findings suggest that weather conditions characterized by reduced visibility or more demanding driving conditions were accompanied by lower reported WVC frequencies, although the mechanisms underlying these associations cannot be determined from the present dataset. One possible explanation is that adverse weather conditions influence both wildlife activity and human travel behavior, thereby reducing opportunities for wildlife-vehicle encounters, while prolonged dry periods may also alter wildlife movement or habitat use. However, these mechanisms were not directly evaluated in the present study. The absence of independent effects of daily mean temperature and atmospheric pressure after adjustment for the remaining hydro-meteorological variables further indicates that individual weather variables may provide limited explanatory power when considered separately. Instead, the results suggest that combinations of environmental conditions and weather-related events may better explain temporal variation in reported WVC frequency. These findings are consistent with previous studies indicating that WVC occurrence is influenced by multiple interacting environmental factors rather than isolated meteorological variables [24,36].

The regional analyses provided additional insight into the spatial consistency of the observed environmental associations. Although the magnitude of several estimated effects varied among regions, their direction was generally consistent with the national analyses, suggesting that the identified relationships were relatively robust across Serbia. However, nearly all nominally significant regional associations lost statistical significance after applying the false discovery rate correction for multiple comparisons. This outcome is likely attributable to the reduced sample size and lower number of environmental events available after stratifying the national dataset into four regional subsets, resulting in wider confidence intervals and reduced statistical power. Consequently, the regional analyses should be interpreted as exploratory rather than confirmatory. The broadly consistent direction of the estimated associations indicates that the national findings are unlikely to be driven by a single geographic region. Differences in climate, topography, habitat composition, wildlife communities, and road-network characteristics among Serbian regions may contribute to the observed spatial variability [40,41], although evaluating the relative importance of these factors requires more detailed ecological and traffic data than were available in the present study.

Several limitations should be considered when interpreting the present findings. The available database did not contain species-specific information, preventing assessment of whether different taxonomic groups respond differently to environmental conditions. Consequently, the reported associations represent aggregated patterns across all recorded WVCs, although previous studies have shown that species differ considerably in their movement behavior, habitat use, and susceptibility to road mortality [10,18,21,25,27,42,43]. Comprehensive traffic exposure data, such as traffic volume, vehicle speed, or vehicle kilometers traveled, were not available at the national scale throughout the study period. Therefore, the analyses focused on reported WVC frequency rather than collision risk, and the observed associations should not be interpreted as direct evidence of changes in wildlife movement or driver behavior. Furthermore, the meteorological data were derived from gridded datasets without the available road characteristics datasets, limiting the incorporation of local road characteristics and landscape features, such as road geometry that may also influence WVC occurrence [44,45]. Although the event-based analytical framework identified statistically robust associations between several environmental conditions and reported WVC frequency, the analyses remain observational and do not establish causal relationships. Future studies should combine species-specific collision records, higher-resolution traffic and environmental data, and detailed spatial information to better understand the mechanisms underlying the observed associations.

## 5. Conclusions

This paper examined the associations between temperature extremes, abrupt temperature changes, and broader hydro-meteorological conditions and reported wildlife-vehicle collision frequency in Serbia using an event-based approach. The results demonstrate that environmental influences on reported WVC frequency are complex and temporally dependent. Prolonged cold episodes were associated with lower reported WVC frequencies, whereas heat episodes showed no significant associations. In contrast, abrupt cool-down shocks were associated with a short-term increase in reported WVC frequency on the day they occurred. Moderate and heavy precipitation, snow-day conditions, and prolonged dry periods were also associated with lower reported WVC frequencies, while daily mean temperature and atmospheric pressure showed no independent associations in the multivariable models. Although the magnitude of the estimated effects varied regionally, the regional analyses broadly supported the national findings, and no regional associations remained statistically significant after correction for multiple testing.

The findings demonstrate the value of considering environmental events across multiple temporal scales when investigating wildlife-vehicle collisions and provide a foundation for future studies and evidence-based mitigation strategies aimed at improving both road safety and wildlife conservation.

## Figures and Tables

**Table 1:** Descriptive statistics of the municipality-day analytical dataset (2016–2025).

Variable	Unit	Mean ± SD	Median (IQR)	Minimum	Maximum
Daily reported WVC count	count	0.069 ± 0.277	0 (0–0)	0	4
Mean air temperature	°C	12.22 ± 9.33	12.16 (4.67–19.90)	−17.64	34.81
Daily precipitation	mm	1.98 ± 4.34	0.15 (0.00–1.94)	0.00	162.16
Atmospheric pressure	kPa	97.71 ± 2.79	98.19 (95.85–100.08)	89.42	103.38

**Table 2:** Monthly effects on reported WVC frequency estimated using the final Poisson regression model.

Month	IRR	Clustered CI Low	Clustered CI High	Clustered SE	Clustered *p*
January	1.000	1.000	1.000	-	-
February	1.017	0.900	1.149	0.059	0.781
March	1.166	1.040	1.306	0.055	0.010
April	1.493	1.329	1.677	0.056	<0.001
May	1.259	1.051	1.509	0.088	0.015
June	1.087	0.944	1.251	0.068	0.235
July	1.036	0.893	1.202	0.072	0.628
August	1.334	1.183	1.505	0.058	<0.001
September	1.024	0.913	1.147	0.055	0.677
October	1.256	1.067	1.478	0.079	0.008
November	1.447	1.316	1.590	0.046	<0.001
December	1.378	1.239	1.533	0.052	<0.001

**Table 3:** Seasonal effects on reported WVC frequency across the four study regions estimated using the final Poisson regression models.

Season	Vojvodina	Belgrade	Central-West	South-East
Spring	1.27 (1.03–1.57) *	1.09 (1.09–1.09) *	1.10 (0.93–1.29)	1.14 (0.91–1.43)
Summer	1.16 (0.94–1.44)	0.93 (0.93–0.93) *	0.93 (0.80–1.08)	1.03 (0.84–1.27)
Autumn	1.10 (0.94–1.30)	1.02 (1.02–1.02) *	1.18 (1.00–1.38) *	1.09 (0.93–1.27)

Winter was used as the reference season within each regional Poisson regression model (IRR = 1.00). * *p* < 0.05.

## Data Availability

The original contributions presented in this study are included in the article/Supplementary Materials. Further inquiries can be directed to the corresponding author.

## Supplementary Materials

The following supporting information can be downloaded at https://www.mdpi.com/article/10.3390/ani16162472/s1, Table S1. Descriptive characteristics of temperature-episode phases used in the national analyses; Table S2. Associations between temperature-episode phases and reported WVC frequency estimated using the final Poisson regression model; Table S3. Descriptive characteristics of temperature-shock phases used in the national analyses; Table S4. Associations between temperature-shock phases and reported WVC frequency estimated using the Poisson regression model; Table S5. Descriptive characteristics of hydro-meteorological predictors included in the national analyses; Table S6. Associations between hydro-meteorological conditions and reported WVC frequency estimated using the Poisson regression model; Table S7. Comparison of shared predictor estimates between the primary snow-proxy model and the alternative temperature-by-precipitation interaction model; Table S8. Model-fit comparison between the primary snow-day proxy model and the alternative temperature-by-precipitation interaction model; Table S9. Regional associations between temperature-episode phases and reported WVC frequency estimated using Poisson regression models, including false discovery rate (FDR) significance levels; Table S10. Regional associations between temperature-shock phases and reported WVC frequency estimated using Poisson regression models, including false discovery rate (FDR) significance levels; Table S11. Regional associations between hydro-meteorological conditions and reported WVC frequency estimated using Poisson regression models; Table S12. Comparison of Poisson and Negative Binomial regression models used to evaluate model fit and overdispersion; Table S13. Summary of diagnostic statistics for the final Poisson regression model; Table S14. Summary of Ljung–Box tests evaluating temporal autocorrelation of residuals from the final Poisson regression model; Figure S1. Observed and predicted frequencies of daily WVC counts under the final Poisson regression model.
