# Supplementary material for: Event-Based Analysis of Wildlife-Vehicle Collisions Under Temperature Extremes and Weather Variability
Source: Animals (Basel). 2026 Aug 8;16(16):2472. doi: 10.3390/ani16162472 (PMC13508847; doi:10.3390/ani16162472)
Supplement: Supplementary file 1 [file animals-16-02472-s001.zip › animals-4462674-supplementary.pdf]

## National analyses – temperature episodes

**Table S1**. Descriptive characteristics of temperature-episode phases used in the national analyses

| Episode phase | Municipality-days | Reported WVCs | Mean daily WVC | Zero-count observations (%) |
|---|---|---|---|---|
| **Heat episode onset** | 220 | 19 | 0.086 | 91.8 |
| **Heat episode middle** | 369 | 26 | 0.070 | 94.3 |
| **Heat episode end** | 220 | 9 | 0.041 | 96.4 |
| **Heat episode (+1 day)** | 220 | 15 | 0.068 | 93.2 |
| **Heat episode (+2 day)** | 215 | 14 | 0.065 | 94.0 |
| **Cold episode onset** | 278 | 5 | 0.018 | 98.2 |
| **Cold episode middle** | 429 | 17 | 0.040 | 96.3 |
| **Cold episode end** | 278 | 10 | 0.036 | 96.4 |
| **Cold episode (+1 day)** | 278 | 20 | 0.072 | 93.5 |
| **Cold episode (+2 day)** | 256 | 17 | 0.066 | 93.8 |

Municipality-days represent the number of municipality-specific daily observations classified into each episode phase across the study period.

**Table S2**. Associations between temperature-episode phases and reported WVC frequency estimated using the final Poisson regression model

| Episode phase | IRR | Clustered CI low | Clustered CI high | Clustered SE | Clustered *p* | Conventional *p* |
|---|---|---|---|---|---|---|
| **Heat episode onset** | 1.218 | 0.724 | 2.048 | 0.252 | 0.442 | 0.396 |
| **Heat episode middle** | 1.068 | 0.639 | 1.785 | 0.249 | 0.795 | 0.745 |
| **Heat episode end** | 0.579 | 0.258 | 1.298 | 0.391 | 0.175 | 0.102 |
| **Heat episode (+1 day)** | 1.055 | 0.674 | 1.653 | 0.217 | 0.807 | 0.837 |
| **Heat episode (+2 days)** | 0.981 | 0.578 | 1.665 | 0.256 | 0.941 | 0.943 |
| **Cold episode onset** | 0.306 | 0.138 | 0.679 | 0.386 | 0.005 | 0.008 |
| **Cold episode middle** | 0.714 | 0.385 | 1.325 | 0.299 | 0.272 | 0.175 |
| **Cold episode end** | 0.619 | 0.338 | 1.135 | 0.293 | 0.115 | 0.133 |
| **Cold episode (+1 day)** | 1.222 | 0.870 | 1.716 | 0.165 | 0.235 | 0.377 |
| **Cold episode (+2 days)** | 1.117 | 0.732 | 1.705 | 0.205 | 0.594 | 0.652 |

## National analyses – temperature shocks

**Table S3**. Descriptive characteristics of temperature-shock phases used in the national analyses

| Shock phase | Municipality-days | Number of recorded WVCs | Mean daily WVC | Zero-count observations (%) | Mean delta temperature |
|---|---|---|---|---|---|
| **No same-day or lagged shock** | 67822 | 4636 | 0.068 | 93.73655 | 0.071 |
| **Warm-up shock, same day** | 4603 | 341 | 0.074 | 93.17836 | 4.173 |
| **Warm-up shock, +1 day** | 4603 | 324 | 0.070 | 93.43906 | 0.816 |
| **Warm-up shock, +2 days** | 4603 | 320 | 0.069 | 93.852 | -0.793 |
| **Cool-down shock, same day** | 4582 | 332 | 0.072 | 93.452 | -5.837 |
| **Cool-down shock, +1 day** | 4581 | 320 | 0.070 | 93.756 | -0.699 |
| **Cool-down shock, +2 days** | 4581 | 337 | 0.073 | 93.124 | 1.182 |

Municipality-days represent the number of municipality-specific daily observations classified into each episode phase across the study period.

**Table S4**. Associations between temperature-shock phases and reported WVC frequency estimated using Poisson regression model

| Shock phase | IRR | Clustered CI low | Clustered CI high | Clustered SE | Clustered *p* | Conventional *p* |
|---|---|---|---|---|---|---|
| **Warm-up shock, same day** | 1.038 | 0.919 | 1.171 | 0.059 | 0.535 | 0.520 |
| **Warm-up shock, +1 day** | 1.019 | 0.907 | 1.144 | 0.056 | 0.747 | 0.752 |
| **Warm-up shock, +2 days** | 0.989 | 0.842 | 1.161 | 0.078 | 0.886 | 0.846 |
| **Cool-down shock, same day** | 1.152 | 1.005 | 1.320 | 0.066 | 0.042 | 0.017 |
| **Cool-down shock, +1 day** | 0.996 | 0.852 | 1.164 | 0.076 | 0.959 | 0.946 |
| **Cool-down shock, +2 days** | 1.057 | 0.951 | 1.176 | 0.051 | 0.288 | 0.325 |

## National analyses – broader hydro-meteorological conditions

**Table S5**. Descriptive characteristics of hydro-meteorological predictors included in the national analyses

| Variable | Category | Municipality-days | Number of recorded WVCs | Mean daily WVC | Zero-count observations (%) |
|---|---|---|---|---|---|
| **Precipitation** | No precipitation | 26130 | 1879 | 0.072 | 93.413 |
| **Precipitation** | Light (>0–5 mm) | 53541 | 3707 | 0.069 | 93.674 |
| **Precipitation** | Moderate (>5–20 mm) | 10747 | 643 | 0.059 | 94.435 |
| **Precipitation** | Heavy (>20 mm) | 907 | 52 | 0.057 | 95.038 |
| **Snow-day proxy** | No | 83747 | 5912 | 0.070 | 93.532 |
| **Snow-day proxy** | Yes | 7578 | 369 | 0.049 | 95.592 |
| **Dry-spell duration** | 0–2 consecutive dry days | 53072 | 3604 | 0.068 | 93.725 |
| **Dry-spell duration** | 3–6 consecutive dry days | 21251 | 1578 | 0.074 | 93.304 |
| **Dry-spell duration** | ≥7 consecutive dry days | 17002 | 1099 | 0.064 | 94.130 |

Municipality-days represent the number of municipality-specific daily observations classified into each episode phase across the study period.

**Table S6**. Associations between hydro-meteorological conditions and reported WVC frequency estimated using Poisson regression model

| Predictor | IRR | Percent change | Clustered CI low | Clustered CI high | Clustered SE | Clustered p | Conventional p |
|---|---|---|---|---|---|---|---|
| **Light precipitation (>0–5 mm)** | 0.988 | -1.222 | 0.928 | 1.051 | 0.030 | 0.687 | 0.706 |
| **Moderate precipitation (>5–20 mm)** | 0.820 | -18.035 | 0.735 | 0.914 | 0.053 | 0.001 | 0.000 |
| **Heavy precipitation (>20 mm)** | 0.760 | -23.987 | 0.590 | 0.980 | 0.123 | 0.036 | 0.057 |
| **Snow-day proxy** | 0.784 | -21.626 | 0.686 | 0.896 | 0.065 | 0.001 | 0.000 |
| **Dry spell (3–6 consecutive days)** | 1.027 | 2.713 | 0.947 | 1.113 | 0.039 | 0.500 | 0.435 |
| **Dry spell (≥7 consecutive days)** | 0.904 | -9.564 | 0.848 | 0.964 | 0.031 | 0.003 | 0.012 |
| **Daily mean temperature** | 1.000 | -0.010 | 0.992 | 1.008 | 0.004 | 0.980 | 0.980 |
| **Atmospheric pressure** | 0.998 | -0.196 | 0.956 | 1.042 | 0.021 | 0.926 | 0.932 |

## Snow day proxy sensitivity analysis

**Table S7**. Comparison of shared predictor estimates between the primary snow-proxy model and the alternative temperature-by-precipitation interaction model

| Variable | Primary IRR | Primary CI low | Primary CI high | Primary $p$ | Alternative IRR | Alternative CI low | Alternative CI high | Alternative $p$ | Absolute IRR difference |
|---|---|---|---|---|---|---|---|---|---|
| **Dry spell (3–6 days)** | 1.027 | 0.947 | 1.113 | 0.500 | 0.983 | 0.904 | 1.068 | 0.668 | 0.044 |
| **Dry spell (≥7 days)** | 0.904 | 0.848 | 0.964 | 0.003 | 0.868 | 0.812 | 0.927 | 0.000 | 0.037 |
| **Temperature** | 1.000 | 0.992 | 1.008 | 0.980 | 1.000 | 0.992 | 1.009 | 0.971 | 0.000 |
| **Pressure** | 0.998 | 0.956 | 1.042 | 0.926 | 0.958 | 0.910 | 1.007 | 0.090 | 0.040 |

**Table S8**. Model-fit comparison between the primary snow-day proxy model and the alternative temperature-by-precipitation interaction model

| Model | No. of observ. | No. of param. | Log likelihood | AIC | BIC | Pearson dispers. | Observed zero count | Expected zero count | Delta AIC | Delta BIC |
|---|---|---|---|---|---|---|---|---|---|---|
| **Prim.** | 91325 | 59 | -20189.9 | 40497.85 | 41053.76 | 1.013396 | 85574 | 85542.88 | 14.14781 | 14.14781 |
| **Alt.** | 91325 | 59 | -20182.9 | 40483.71 | 41039.62 | 1.014703 | 85574 | 85543.81 | 0 | 0 |

Prim - Primary snow-proxy model

Alt - Alternative interaction model

## Regional analyses – temperature episodes

**Table S9**. Regional associations between temperature-episode phases and reported WVC frequency estimated using Poisson regression models, including false discovery rate (FDR) significance levels

| Region | Predi-ctor | IRR | % Δ | Robust CI low | Robust CI high | Robust SE | Robust *p* | FDR adjusted *p* | Sig. raw 0.05 | Sig. after FDR |
|---|---|---|---|---|---|---|---|---|---|---|
| **V** | Heat onset | 1.649 | 64.860 | 0.681 | 3.992 | 0.361 | 0.216 | 0.687 | F | F |
| **V** | Heat middle | 1.377 | 37.659 | 0.541 | 3.501 | 0.381 | 0.434 | 0.806 | F | F |
| **V** | Heat end | 1.165 | 16.474 | 0.360 | 3.771 | 0.480 | 0.762 | 0.866 | F | F |
| **V** | Heat (+1 day) | 0.814 | -18.637 | 0.294 | 2.252 | 0.416 | 0.638 | 0.833 | F | F |
| **V** | Heat (+2 days) | 1.265 | 26.547 | 0.330 | 4.852 | 0.549 | 0.683 | 0.854 | F | F |
| **V** | Cold onset | 0.267 | -73.257 | 0.019 | 3.693 | 1.073 | 0.265 | 0.687 | F | F |
| **V** | Cold middle | 0.373 | -62.680 | 0.102 | 1.367 | 0.530 | 0.113 | 0.687 | F | F |
| **V** | Cold end | 0.566 | -43.421 | 0.126 | 2.549 | 0.615 | 0.390 | 0.781 | F | F |
| **V** | Cold (+1 day) | 1.039 | 3.945 | 0.436 | 2.477 | 0.355 | 0.917 | 0.917 | F | F |
| **V** | Cold (+2 days) | 1.893 | 89.263 | 1.031 | 3.475 | 0.248 | 0.042 | 0.687 | T | F |
| **B** | Heat onset | 1.264 | 26.355 | 0.616 | 2.591 | 0.366 | 0.523 | 0.833 | F | F |
| **B** | Heat middle | 1.382 | 38.159 | 0.566 | 3.375 | 0.456 | 0.478 | 0.806 | F | F |
| **B** | Heat end | 0.649 | -35.083 | 0.203 | 2.077 | 0.593 | 0.467 | 0.806 | F | F |
| **B** | Heat (+1 day) | 0.706 | -29.437 | 0.199 | 2.508 | 0.647 | 0.590 | 0.833 | F | F |
| **B** | Heat (+2 days) | 1.437 | 43.690 | 0.521 | 3.962 | 0.518 | 0.484 | 0.806 | F | F |
| **B** | Cold onset | 0.207 | -79.282 | 0.030 | 1.439 | 0.989 | 0.111 | 0.687 | F | F |
| **B** | Cold middle | 1.422 | 42.176 | 0.795 | 2.542 | 0.296 | 0.235 | 0.687 | F | F |
| **B** | Cold end | 0.415 | -58.497 | 0.119 | 1.442 | 0.636 | 0.166 | 0.687 | F | F |
| **B** | Cold (+1 day) | 1.133 | 13.296 | 0.470 | 2.732 | 0.449 | 0.781 | 0.866 | F | F |

| | | | | | | | | | | |
|---|---|---|---|---|---|---|---|---|---|---|
| **B** | Cold (+2 days) | 1.227 | 22.658 | 0.513 | 2.931 | 0.445 | 0.646 | 0.833 | F | F |
| **CW** | Heat onset | 0.521 | -47.902 | 0.138 | 1.966 | 0.543 | 0.275 | 0.687 | F | F |
| **CW** | Heat middle | 1.166 | 16.646 | 0.173 | 7.863 | 0.780 | 0.850 | 0.872 | F | F |
| **CW** | Heat end | 0.281 | -71.872 | 0.049 | 1.612 | 0.713 | 0.126 | 0.687 | F | F |
| **CW** | Heat (+1 day) | 0.914 | -8.645 | 0.325 | 2.570 | 0.423 | 0.838 | 0.872 | F | F |
| **CW** | Heat (+2 days) | 0.905 | -9.510 | 0.357 | 2.294 | 0.380 | 0.801 | 0.866 | F | F |
| **CW** | Cold onset | 0.290 | -71.041 | 0.024 | 3.513 | 1.020 | 0.270 | 0.687 | F | F |
| **CW** | Cold middle | 0.592 | -40.815 | 0.246 | 1.423 | 0.359 | 0.194 | 0.687 | F | F |
| **CW** | Cold end | 0.847 | -15.285 | 0.287 | 2.498 | 0.442 | 0.720 | 0.862 | F | F |
| **CW** | Cold (+1 day) | 1.695 | 69.533 | 0.633 | 4.543 | 0.403 | 0.238 | 0.687 | F | F |
| **CW** | Cold (+2 days) | 0.571 | -42.868 | 0.131 | 2.488 | 0.601 | 0.388 | 0.781 | F | F |
| **SE** | Heat onset | 1.265 | 26.488 | 0.280 | 5.720 | 0.667 | 0.733 | 0.862 | F | F |
| **SE** | Heat middle | 0.584 | -41.563 | 0.164 | 2.084 | 0.562 | 0.364 | 0.781 | F | F |
| **SE** | Heat end | 0.215 | -78.545 | 0.022 | 2.110 | 1.010 | 0.162 | 0.687 | F | F |
| **SE** | Heat (+1 day) | 1.603 | 60.325 | 0.762 | 3.372 | 0.329 | 0.185 | 0.687 | F | F |
| **SE** | Heat (+2 days) | 0.471 | -52.852 | 0.078 | 2.840 | 0.794 | 0.368 | 0.781 | F | F |
| **SE** | Cold onset | 0.448 | -55.175 | 0.114 | 1.762 | 0.605 | 0.218 | 0.687 | F | F |
| **SE** | Cold middle | 0.441 | -55.903 | 0.148 | 1.318 | 0.484 | 0.125 | 0.687 | F | F |
| **SE** | Cold end | 0.677 | -32.342 | 0.139 | 3.298 | 0.700 | 0.591 | 0.833 | F | F |
| **SE** | Cold (+1 day) | 1.133 | 13.317 | 0.682 | 1.884 | 0.225 | 0.592 | 0.833 | F | F |
| **SE** | Cold (+2 days) | 0.763 | -23.705 | 0.216 | 2.695 | 0.558 | 0.639 | 0.833 | F | F |

V – Vojvodina; B – Belgrade; CW – Central West; SE – South East; T – True, F – False

## Regional analyses – temperature shocks

**Table S10**. Regional associations between temperature-shock phases and reported WVC frequency estimated using Poisson regression models, including false discovery rate (FDR) significance levels

| Region | Predictor | IRR | % Δ | Robust CI low | Robust CI high | Robust SE | Robust *p* | FDR adjusted *p* | Sig. raw 0.05 | Sig. after FDR |
|---|---|---|---|---|---|---|---|---|---|---|
| **V** | Warm-up, same day | 1.128 | 12.822 | 0.824 | 1.544 | 0.128 | 0.383 | 0.907 | F | F |
| **V** | Warm-up, +1 day | 1.227 | 22.702 | 0.900 | 1.674 | 0.127 | 0.158 | 0.758 | F | F |
| **V** | Warm-up, +2 days | 0.918 | -8.169 | 0.608 | 1.388 | 0.169 | 0.631 | 0.907 | F | F |
| **V** | Cool-down, same day | 1.194 | 19.399 | 0.931 | 1.531 | 0.102 | 0.131 | 0.758 | F | F |
| **V** | Cool-down, +1 | 1.020 | 2.035 | 0.638 | 1.631 | 0.192 | 0.920 | 0.924 | F | F |
| **V** | Cool-down, +2 | 0.959 | -4.107 | 0.705 | 1.304 | 0.126 | 0.750 | 0.907 | F | F |
| **B** | Warm-up, same day | 1.038 | 3.813 | 0.821 | 1.313 | 0.120 | 0.755 | 0.907 | F | F |
| **B** | Warm-up, +1 day | 0.977 | -2.291 | 0.782 | 1.221 | 0.114 | 0.838 | 0.915 | F | F |
| **B** | Warm-up, +2 days | 1.140 | 13.994 | 0.908 | 1.431 | 0.116 | 0.259 | 0.907 | F | F |
| **B** | Cool-down, same day | 1.216 | 21.575 | 0.974 | 1.518 | 0.113 | 0.085 | 0.758 | F | F |
| **B** | Cool-down, +1 | 1.120 | 12.044 | 0.891 | 1.408 | 0.117 | 0.330 | 0.907 | F | F |
| **B** | Cool-down, +2 | 1.055 | 5.523 | 0.845 | 1.317 | 0.113 | 0.635 | 0.907 | F | F |
| **CW** | Warm-up, same day | 0.874 | -12.613 | 0.574 | 1.330 | 0.172 | 0.462 | 0.907 | F | F |
| **CW** | Warm-up, +1 day | 0.902 | -9.756 | 0.638 | 1.276 | 0.142 | 0.496 | 0.907 | F | F |
| **CW** | Warm-up, +2 days | 1.057 | 5.718 | 0.643 | 1.739 | 0.203 | 0.794 | 0.907 | F | F |
| **CW** | Cool-down, same day | 0.924 | -7.564 | 0.543 | 1.573 | 0.217 | 0.730 | 0.907 | F | F |
| **CW** | Cool-down, +1 | 0.823 | -17.731 | 0.510 | 1.327 | 0.196 | 0.357 | 0.907 | F | F |
| **CW** | Cool-down, +2 | 1.218 | 21.762 | 0.915 | 1.620 | 0.117 | 0.143 | 0.758 | F | F |
| **SE** | Warm-up, same day | 1.099 | 9.883 | 0.846 | 1.427 | 0.116 | 0.436 | 0.907 | F | F |

| | | | | | | | | | | |
|---|---|---|---|---|---|---|---|---|---|---|
| **SE** | Warm-up, +1 day | 0.975 | -2.466 | 0.814 | 1.169 | 0.080 | 0.762 | 0.907 | F | F |
| **SE** | Warm-up, +2 days | 0.869 | -13.141 | 0.639 | 1.181 | 0.136 | 0.327 | 0.907 | F | F |
| **SE** | Cool-down, same day | 1.237 | 23.710 | 1.050 | 1.457 | 0.072 | 0.017 | 0.396 | T | F |
| **SE** | Cool-down, +1 | 0.989 | -1.072 | 0.772 | 1.267 | 0.109 | 0.924 | 0.924 | F | F |
| **SE** | Cool-down, +2 | 1.020 | 2.010 | 0.880 | 1.182 | 0.065 | 0.767 | 0.907 | F | F |

V – Vojvodina; B – Belgrade; CW – Central West; SE – South East; T – True, F – False

## Regional analyses – broader hydro-meteorological conditions

**Table S11**. Regional associations between hydro-meteorological conditions and reported WVC frequency estimated using Poisson regression models

| Region | Predictor | IRR | % Δ | Robust CI low | Robust CI high | Robust SE | Robust p | FDR adjusted p | Sig. raw 0.05 | Sig. after FDR |
|---|---|---|---|---|---|---|---|---|---|---|
| **V** | Light precip. (0-5 mm) | 0.997 | -0.278 | 0.835 | 1.191 | 0.073 | 0.971 | 0.971 | F | F |
| **V** | Moderate precip. (5-20 mm) | 0.788 | -21.230 | 0.561 | 1.105 | 0.138 | 0.135 | 0.475 | F | F |
| **V** | Heavy precip. (>20 mm) | 0.442 | -55.769 | 0.138 | 1.413 | 0.475 | 0.137 | 0.475 | F | F |
| **V** | Snow-day proxy | 0.731 | -26.871 | 0.502 | 1.065 | 0.154 | 0.088 | 0.468 | F | F |
| **V** | Dry spell (3-6 days) | 1.074 | 7.426 | 0.861 | 1.340 | 0.090 | 0.458 | 0.706 | F | F |
| **V** | Dry spell (≥7 days) | 0.895 | -10.483 | 0.728 | 1.100 | 0.084 | 0.237 | 0.632 | F | F |
| **V** | Daily mean temperature | 1.002 | 0.236 | 0.995 | 1.010 | 0.003 | 0.478 | 0.706 | F | F |
| **V** | Atmospheric pressure | 0.927 | -7.297 | 0.873 | 0.985 | 0.025 | 0.022 | 0.356 | T | F |
| **B** | Light precip. (0-5 mm) | 1.024 | 2.378 | 0.891 | 1.176 | 0.071 | 0.740 | 0.876 | F | F |
| **B** | Moderate precip. (5-20 mm) | 0.889 | -11.069 | 0.718 | 1.101 | 0.109 | 0.283 | 0.646 | F | F |
| **B** | Heavy precip. (>20 mm) | 0.983 | -1.740 | 0.558 | 1.729 | 0.288 | 0.951 | 0.971 | F | F |
| **B** | Snow-day proxy | 0.895 | -10.493 | 0.678 | 1.181 | 0.142 | 0.434 | 0.706 | F | F |
| **B** | Dry spell (3-6 days) | 1.060 | 6.036 | 0.920 | 1.222 | 0.072 | 0.418 | 0.706 | F | F |
| **B** | Dry spell (≥7 days) | 0.942 | -5.757 | 0.801 | 1.109 | 0.083 | 0.474 | 0.706 | F | F |

| | | | | | | | | | | |
|---|---|---|---|---|---|---|---|---|---|---|
| **B** | Daily mean temperature | 0.992 | -0.849 | 0.977 | 1.006 | 0.008 | 0.260 | 0.640 | F | F |
| **B** | Atmospheric pressure | 1.032 | 3.225 | 0.944 | 1.128 | 0.045 | 0.485 | 0.706 | F | F |
| **CW** | Light precip. (0-5 mm) | 1.015 | 1.492 | 0.825 | 1.248 | 0.085 | 0.867 | 0.956 | F | F |
| **CW** | Moderate precip. (5-20 mm) | 0.817 | -18.316 | 0.612 | 1.091 | 0.118 | 0.138 | 0.475 | F | F |
| **CW** | Heavy precip. (>20 mm) | 0.883 | -11.697 | 0.555 | 1.405 | 0.190 | 0.537 | 0.716 | F | F |
| **CW** | Snow-day proxy | 0.655 | -34.547 | 0.482 | 0.888 | 0.125 | 0.014 | 0.356 | T | F |
| **CW** | Dry spell (3-6 days) | 0.953 | -4.689 | 0.770 | 1.180 | 0.087 | 0.602 | 0.771 | F | F |
| **CW** | Dry spell (≥7 days) | 0.913 | -8.700 | 0.784 | 1.063 | 0.062 | 0.193 | 0.562 | F | F |
| **CW** | Daily mean temperature | 1.003 | 0.303 | 0.979 | 1.028 | 0.010 | 0.775 | 0.886 | F | F |
| **CW** | Atmospheric pressure | 1.022 | 2.159 | 0.946 | 1.103 | 0.031 | 0.521 | 0.716 | F | F |
| **SE** | Light precip. (0-5 mm) | 0.950 | -4.983 | 0.844 | 1.070 | 0.053 | 0.356 | 0.706 | F | F |
| **SE** | Moderate precip. (5-20 mm) | 0.811 | -18.904 | 0.653 | 1.007 | 0.096 | 0.056 | 0.451 | F | F |
| **SE** | Heavy precip. (>20 mm) | 0.696 | -30.384 | 0.484 | 1.001 | 0.161 | 0.051 | 0.451 | F | F |
| **SE** | Snow-day proxy | 0.794 | -20.604 | 0.613 | 1.029 | 0.114 | 0.075 | 0.468 | F | F |
| **SE** | Dry spell (3-6 days) | 1.033 | 3.267 | 0.877 | 1.215 | 0.072 | 0.666 | 0.820 | F | F |
| **SE** | Dry spell (≥7 days) | 0.893 | -10.657 | 0.760 | 1.050 | 0.071 | 0.148 | 0.475 | F | F |
| **SE** | Daily mean temperature | 0.999 | -0.058 | 0.982 | 1.017 | 0.008 | 0.942 | 0.971 | F | F |

| | | | | | | | | | | |
|---|---|---|---|---|---|---|---|---|---|---|
| **SE** | Atmospheric pressure | 1.027 | 2.723 | 0.946 | 1.116 | 0.036 | 0.480 | 0.706 | F | F |

V – Vojvodina; B – Belgrade; CW – Central West; SE – South East; T – True, F – False

## Poisson and Negative Binomial model comparisons

**Table S12**. Comparison of Poisson and Negative Binomial regression models used to evaluate model fit and overdispersion

| Metric | Poisson | Negative Binomial |
|---|---|---|
| **Number of observations** | 91325 | 91325 |
| **Number of estimated parameters** | 63 | 64 |
| **Log-likelihood** | -20187.4 | -20186.3 |
| **AIC** | 40500.83 | 40500.59 |
| **BIC** | 41094.43 | 41103.61 |
| **Observed zero count** | 85574 | 85574 |
| **Expected zero count** | 85543.06 | 85569.76 |
| **Observed zero percentage** | 93.70271 | 93.70271 |
| **Expected zero percentage** | 93.66883 | 93.69807 |
| **Pearson chi-square** | 92333.41 | |
| **Pearson dispersion** | 1.01174 | |
| **Estimated NB2 alpha** | | 0.066308 |
| **Likelihood-ratio statistic** | | 2.246448 |
| **Conventional LR p-value** | | 0.133921 |
| **Boundary-corrected LR p-value** | | 0.066961 |

**Table S13**. Summary of diagnostic statistics for the final Poisson regression model

| Metric | Value |
|---|---|
| **Number of observations** | 91325 |
| **Residual degrees of freedom** | 91262 |
| **Pearson chi-square** | 92333.41 |
| **Pearson dispersion** | 1.01174 |
| **Observed zero count** | 85574 |
| **Observed zero percentage** | 93.70271 |
| **Predicted zero count** | 85543.06 |
| **Predicted zero percentage** | 93.66883 |
| **Mean randomized quantile residual** | -0.00583 |
| **SD randomized quantile residual** | 1.002043 |
| **Shapiro-Wilk statistic** | 0.99929 |

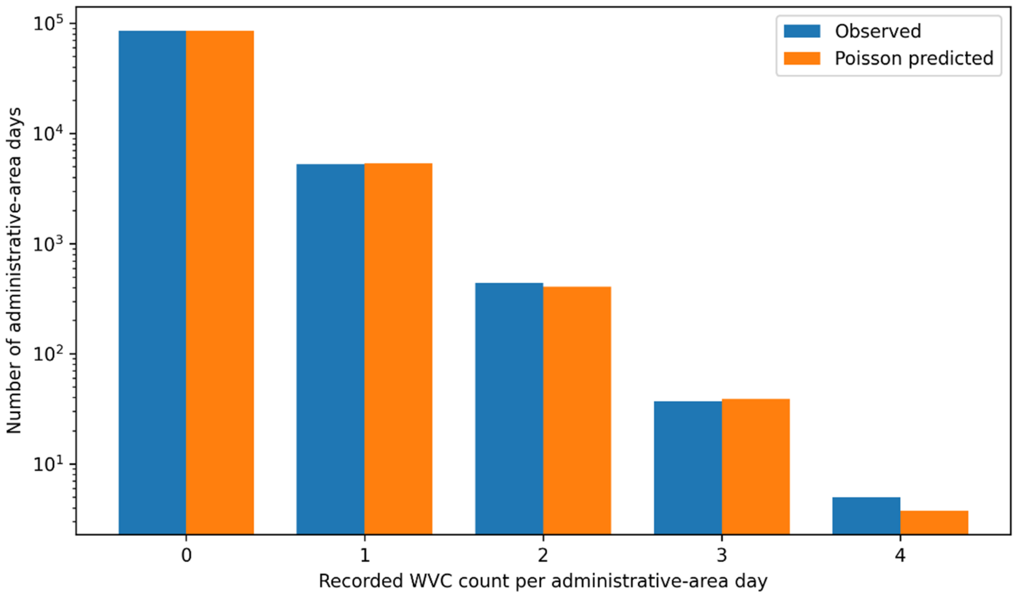


**Figure S1**. Observed and predicted frequencies of daily WVC counts under the final Poisson regression model

**Table S14**. Summary of Ljung-Box tests evaluating temporal autocorrelation of residuals from the final Poisson regression model

| Residual type | Lag days | Number of areas | Areas raw p <0.05 | Areas FDR sig. | Minimum raw p | Median raw p | Minimum FDR p | Median FDR p |
|---|---|---|---|---|---|---|---|---|
| **Deviance residual** | 7 | 25 | 4 | 1 | <0.001 | 0.332218 | 0.002485 | 0.63888 |
| **Deviance residual** | 14 | 25 | 5 | 2 | 0.000149 | 0.254655 | 0.003728 | 0.489721 |
| **Deviance residual** | 30 | 25 | 5 | 3 | <0.001 | 0.311177 | <0.001 | 0.579494 |
| **Pearson residual** | 7 | 25 | 4 | 2 | <0.001 | 0.438017 | <0.001 | 0.758269 |
| **Pearson residual** | 14 | 25 | 2 | 2 | <0.001 | 0.299075 | <0.001 | 0.575145 |
| **Pearson residual** | 30 | 25 | 3 | 3 | <0.001 | 0.287531 | <0.001 | 0.552944 |
| **Randomized quantile residual** | 7 | 25 | 0 | 0 | 0.079717 | 0.387188 | 0.587367 | 0.744593 |
| **Randomized quantile residual** | 14 | 25 | 0 | 0 | 0.097186 | 0.528069 | 0.796346 | 0.895795 |
| **Randomized quantile residual** | 30 | 25 | 0 | 0 | 0.073382 | 0.399724 | 0.703426 | 0.720031 |
